# Omega-3 Polyunsaturated Fatty Acids Prevent *Toxoplasma gondii* Infection by Inducing Autophagy via AMPK Activation

**DOI:** 10.3390/nu11092137

**Published:** 2019-09-06

**Authors:** Jae-Won Choi, Jina Lee, Jae-Hyung Lee, Byung-Joon Park, Eun Jin Lee, Soyeon Shin, Guang-Ho Cha, Young-Ha Lee, Kyu Lim, Jae-Min Yuk

**Affiliations:** 1 Department of Infection Biology, College of Medicine, Chungnam National University, Daejeon 35015, Korea; 2 Department of Medical Science, College of Medicine, Chungnam National University, Daejeon 35015, Korea; 3 Infection Control Convergence Research Center, College of Medicine, Chungnam National University, Daejeon 35015, Korea; 4 Department of Biochemistry, College of Medicine, Chungnam National University, Daejeon 35015, Korea

**Keywords:** omega 3 polyunsaturated fatty acid, autophagy, AMP-activated protein kinase, Fat-1 transgenic mice, *Toxoplasma gondii*, bone marrow-derived macrophages

## Abstract

Omega-3 polyunsaturated fatty acids (ω3-PUFAs) have potential protective activity in a variety of infectious diseases, but their actions and underlying mechanisms in *Toxoplasma gondii* infection remain poorly understood. Here, we report that docosahexaenoic acid (DHA) robustly induced autophagy in murine bone marrow-derived macrophages (BMDMs). Treatment of *T. gondii*-infected macrophages with DHA resulted in colocalization of *Toxoplasma* parasitophorous vacuoles with autophagosomes and reduced intracellular survival of *T. gondii*. The autophagic and anti-*Toxoplasma* effects induced by DHA were mediated by AMP-activated protein kinase (AMPK) signaling. Importantly, BMDMs isolated from Fat-1 transgenic mice, a well-known animal model capable of synthesizing ω3-PUFAs from ω6-PUFAs, showed increased activation of autophagy and AMPK, leading to reduced intracellular survival of *T. gondii* when compared with wild-type BMDMs. Moreover, Fat-1 transgenic mice exhibited lower cyst burden in the brain following infection with the avirulent strain ME49 than wild-type mice. Collectively, our results revealed mechanisms by which endogenous ω3-PUFAs and DHA control *T. gondii* infection and suggest that ω3-PUFAs might serve as therapeutic candidate to prevent toxoplasmosis and infection with other intracellular protozoan parasites.

## 1. Introduction

Omega-3 polyunsaturated fatty acids (ω3-PUFAs) are essential nutrients for human health and body homeostasis. Growing evidence strongly suggests that dietary ω3-PUFAs, especially docosahexaenoic acid (DHA) and eicosapentaenoic acid (EPA), have diverse biological activities, including anti-inflammatory, anti-cancer, and anti-oxidant activities, and may play pivotal roles in inflammatory diseases, allergic diseases, cancer, cardiovascular diseases, and other chronic diseases [1,2,3]. Transgenic mice enriched ω3-PUFAs by introducing the *Caenorhabditis elegans* fat-1 gene encoding an ω3-fatty acid desaturase (Fat-1 transgenic mice), which are able to produce ω3-PUFAs from ω6-PUFAs [4], are protected from inflammation-associated diseases [5,6,7], while Fat-1 transgenic mice and macrophages exhibited impaired host resistance to tuberculosis with enhanced bacterial loads and reduced inflammatory responses [8]. Although the immunomodulatory properties of ω3-PUFAs in infectious diseases, including parasitic, bacterial, and viral infections, have been elaborately demonstrated [9,10], whether ω3-PUFAs are beneficial or harmful in the activation of host-protective immunity against infectious agents remains poorly understood.

*Toxoplasma gondii* can infect a broad range of warm-blooded animals, including avian and mammalian species, and is a major protozoan parasite that affects approximately one-third of the global human population [11]. Although immunocompetent people generally remain asymptomatic, *Toxoplasma* infection in immunocompromised and congenitally infected humans leads to toxoplasmosis, with high morbidity and mortality rates [11,12]. Because of potential side effects of pyrimethamine, such as bone marrow suppression and liver toxicity, there is an urgent need for effective but less-damaging therapeutic agents [13]. *T. gondii* is capable of invading all nucleated cells and the parasite can reside and grow in cells through the formation of parasitophorous vacuoles (PVs). In addition to PV formation, *T. gondii* has evolved multiple strategies to escape host immune defense reactions, enabling persistence and replication in even innate immune cells, including human dendritic cells, macrophages, and neutrophils [14,15,16]. In *Toxoplasma* infection, lipoxin A_4_, a 5-lipoxygenase-derived eicosanoid mediator derived from the ω6-PUFA arachidonic acid, induces increased tissue cyst burden and reduces lethality associated with encephalitis, which is closely associated with the attenuation of interleukin 12 (IL-12) and interferon gamma (IFN-γ) generation [17]. Despite these findings, the immunomodulatory properties and underlying mechanisms of ω3-PUFAs remain unclear.

Autophagy, a conserved homeostatic process, is crucial for the elimination of cellular components such as damaged organelles and long-lived or misfolded proteins through the fusion of autophagosomes and lysosomes in response to various cellular stresses [18]. Autophagy is tightly regulated by the coordinated action of various autophagy-related (Atg) proteins and autophagy-regulating kinases in five distinct steps: initiation, nucleation, expansion, fusion, and degradation. The UNC-51-like kinase 1/2 (ULK1/2) complex (the mammalian ortholog of yeast Atg1) plays a central role in the initiation of autophagy, which is activated by AMP-activated protein kinase (AMPK) and inhibited by mammalian target of rapamycin complex 1 (mTORC1). Importantly, numerous studies have demonstrated the essential role of autophagy in controlling host protective immunity against a broad range of infectious agents, including intracellular bacteria, viruses, and protozoa [19,20]. Although *T. gondii* has various strategies to evade host immune responses, recent studies have suggested that canonical and non-canonical autophagic processes contribute to parasite recognition by the host immune system, leading to intracellular restriction of *T. gondii* in hematopoietic and nonhematopoietic cells [21].

In the present study, we demonstrated that ω3-PUFAs exert potent anti-*Toxoplasma* activities via autophagy activation using primary murine macrophages and Fat-1 transgenic mice. We found that the AMPK signaling pathway is required for the enhancement of autophagy activation in DHA-treated or Fat-1-expressing macrophages. Our findings suggest that ω3-PUFAs are therapeutic candidates for the treatment of *Toxoplasma* infection.

## 2. Materials and Methods

### 2.1. Mice and Cell Culture

Wild-type (WT) C57BL/6 mice were purchased from Koatech (Pyeongtaek, Korea). Fat-1 transgenic mice were kindly provided by Dr. Jing X. Kang (Department of Medicine, Massachusetts General Hospital and Harvard Medical School, USA). Murine bone marrow-derived macrophages (BMDMs) were differentiated for 5–7 days in medium containing macrophage colony-stimulating factor, as described previously [22]. The human retinal pigment epithelial cell line ARPE-19 was purchased from the American Type Culture Collection (Manassas, VA, USA) and cells were grown in Dulbecco’s modified Eagle’s medium (DMEM, Life Technologies, Grand Island, NY, USA) supplemented with 10% fetal bovine serum (FBS, Gibco BRL, Grand Island, NY, USA) or with nutrient mixture F-12, 10% FBS, and 1% antibiotic-antimycotic (Anti-Anti, Gibco BRL). ARPE-19 cells were passaged using 0.25% trypsin-EDTA (Life Technologies, Carlsbad, CA, USA) every 2–3 days.

### 2.2. Parasite Preparation

Tachyzoites of *T. gondii* RH strain were maintained in ARPE-19 cells at 37 °C in an atmosphere with 5% CO_2_ and passaged biweekly in DMEM with 10% FBS, nutrient mixture F-12, and antibiotics. Tachyzoites expressing green fluorescent protein (GFP-RH strain) were kindly provided by Dr. Yoshifumi Nishikawa (Obihiro University of Agriculture and Veterinary Medicine, Japan). Bradyzoites of *T. gondii* strain ME49 were harvested from the brains of BALB/cAnHsd mice that had been inoculated with 50 cysts, and were maintained every 3 weeks.

### 2.3. Experimental Murine Toxoplasmosis Model

To establish an in-vivo toxoplasmosis model, mice were intraperitoneally injected with 40 cysts of strain ME49. Cysts were obtained from brain homogenates 3 weeks after infection. Genomic DNA was extracted from brain homogenates using the G-DEX^TM^ IIc for Cell/Tissue Genomic DNA Extraction Kit (iNtRON Biotechnology, Seongnam-Si, Korea) and the *T. gondii*-specific gene B1 was amplified using specific primers (forward: 5′-GGAACTGCATCCGTTCATGAG-3′; reverse: 5′-TCTTTAAAGCGTTCGTGGTC-3′).

### 2.4. Ethics Statement

Animal experimental procedures were approved by the Institutional Animal Care and Use Committee (IACUC) from Chungnam National University (CNU-00706) and conformed to the National Institutes of Health guidelines. The animals had access to standard rodent food and water ad libitum and were housed (maximum of 5 animals per cage) in sawdust-lined cages in an air-conditioned environment under a 12-h light/dark cycle. Animal husbandry was provided by the staff of the IACUC under the guidance of supervisors who are certified Animal Technologists and by the staff of the Animal Core Facility. Veterinary care was provided by IACUC faculty members and veterinary residents at the Chungnam National University School of Medicine.

### 2.5. Reagents and Antibodies

For in vitro experiments, DHA (90310) was purchased from Cayman Chemical (Ann Arbor, MI, USA). Compound C (171260) was purchased from Calbiochem (San Diego, CA, USA). 3-methyladenine (3-MA), Baf-A1, STO-609, and DMSO (added to macrophages cultures at 0.05% (*v*/*v*) and used as a solvent control) were from Sigma-Aldrich (Saint Louis, MO, USA). Specific antibodies against phospho-AMPKα (2535), phospho-acetyl-CoA carboxylase (3661), phospho-CAMKK2 (12818), phospho-LKB1 (3482) were purchased from Cell Signaling. LC3 (L8918) was purchased from Sigma-Aldrich. Antibody against β-tubulin (ab6046) was purchased from Abcam (Burlingame, CA, USA). Antibody against TP3 (sc-52255) was purchased from Santa Cruz (Santa Cruz, CA, USA). All other reagents were purchased from Sigma-Aldrich, unless otherwise indicated.

### 2.6. RNA Extraction, Real-Time Quantitative Polymerase Chain Reaction (PCR), Semi-Quantitative Reverse Transcription-PCR, and Western Blot Analysis

RNA extraction, real-time quantitative polymerase chain reaction (PCR), and semi-quantitative Reverse Transcription-PCR were conducted as described previously [22]. The sequences of the primers used were as follows: Sag1 (forward: 5′-ATCGCCTGAGAAGCATCACT-3′; reverse: 5′- GCGAAAATGGAAACGTGACT-3′), β-actin (forward: 5′- TCATGAAGTGTGACGTTGACATCCGT-3′; reverse: 5′-CCTAGAAGCATTTGCGGTGCACGATG-3′).

For Western blot analysis, cells were collected and lysed in PRO-PREP (iNtRON Biotechnology) containing a set of phosphatase inhibitors. Protein concentrations were determined using a BCA assay kit. Proteins (30 μg) were immediately heated at 100 °C for 5 min. Each sample was subjected to sodium dodecyl sulfate polyacrylamide gel electrophoresis on a gel containing 12% (*w*/*v*) acrylamide under reducing conditions. Then, the proteins were transferred to polyvinylidene fluoride membranes (Millipore, Billerica, MA, USA), which were blocked with 5% skim milk. Membranes were developed using a chemiluminescence assay kit (Millipore) and analyzed using an Chemiluminescence Imaging System (Davinch-K, Seoul, Korea). Data were analyzed using Alliance Mini HD6 (UVitec, Cambridge, MA, USA).

### 2.7. Immunofluorescence and Confocal Microscopy

To analyze intracellular *T. gondii* proliferation, BMDMs were cultured in 24-well plates on 22-mm glass coverslips and infected with the strain GFP-RH at the indicated multiplicity of infection for the indicated periods. Cells were washed with phosphate-buffered saline (PBS), fixed with 4% paraformaldehyde in PBS for 10 min, and permeabilized with 0.25% Triton X-100 in PBS for 10 min. The cytosol and nuclei were stained with Texas Red^®^-X phalloidin (Life Technologies Corporation) and 4′,6-diamidino-2-phenylindole (DAPI, Sigma), respectively. Coverslips were mounted on the glass slides with Fluoromount-G (SouthernBiotech). Fluorescence images were acquired with a confocal laser-scanning microscope (Leica TCS-SP6, Seoul, Korea). Immunofluorescence analysis of endogenous LC3 puncta, colocalization of LC3 with GFP-RH, and colocalization of LC3 with lysosome-associated membrane glycoprotein 1 (LAMP1) was carried out as described previously [23].

### 2.8. Adenovirus Production

Adenoviral vectors were used for target gene overexpression or mutation. Adenoviruses (Ad) encoding GFP (Ad-GFP), dominant-negative AMPK (Ad-DN-AMPK), and constitutively active AMPK (Ad-CA-AMPK) were kindly provided by Dr. Hueng-Sik Choi (Chonnam National University, Gwangju, Republic of Korea). Ad-DN-LKB1 and Ad-LKB1 were kindly provided by Dr. Don-kyu Kim (Chonnam National University) [24]. Large-scale Ad production was achieved as described previously [25,26].

### 2.9. Statistical Analysis

Differences between independent experimental data were analyzed by using a two-tailed Student’s *t*-test and are presented as the means ± standard deviation (SD) or the means ± standard error of the mean (SEM). Differences were regarded significant at *p* value under 0.05. The number of cysts in brain homogenates was graphed using Graphpad Prism (GraphPad Software Inc., San Diego, CA, USA).

## 3. Results

### 3.1. Endogenous Omega-3 Polyunsaturated Fatty Acids (ω3-PUFAs) Contribute to the Activation of Antiparasitic Responses against Toxoplasma Infection

**Figure 1:**
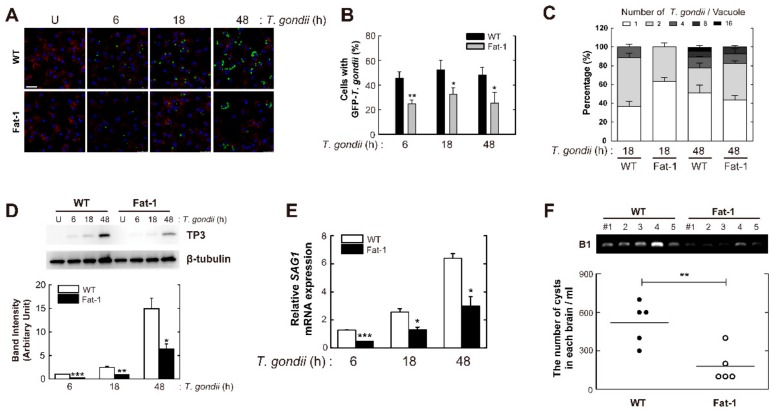
Endogenous omega-3 polyunsaturated fatty acids (ω-3 PUFAs) are required for the inhibition of intracellular *T. gondii* proliferation in macrophages. (**A**–**C**) Bone marrow-derived macrophages (BMDMs) from wild-type (WT) and Fat-1 transgenic mice were infected with *T. gondii* tachyzoites expressing green fluorescent protein (GFP-RH strain) (Multiplicity of infection (MOI) = 1) for the indicated time periods. (**A**) After fixation of cells, F-actin in the cytoskeleton (red) and nuclei (blue) were stained with Texas Red^®^-X phalloidin and 4′,6-diamidino-2-phenylindole (DAPI), respectively. The number of GFP-RH strain was analyzed by confocal microscopy. (**B** and **C**) GFP-RH-infected cells (**B**) and GFP-RH strain per vacuole (**C**) were counted. Scale bar = 25 μm. (**D** and **E**) BMDMs from WT and Fat-1 transgenic mice were infected with *T. gondii* RH strain (MOI = 1) for the indicated time periods. (**D**) Immunoblot analysis was performed to determine protein expression of TP3 and β-tubulin. A representative gel from three independent experiments with similar results is shown (top), Densitometry analysis (bottom) (**E**) *SAG1* mRNA expression was evaluated by quantitative polymerase chain reaction (qPCR) analysis. (**F**) WT and Fat-1 transgenic mice (*n* = 5 per group) were infected with 40 cysts of *T. gondii* ME49 strain (intraperitoneal injection). After 3 weeks, *B1* gene expression in brain homogenates was detected by PCR analysis (top). Cysts in the brain were counted under a microscope (bottom). Data are representative of three independent experiments and are presented as means ± SD. * *p* < 0.05, ** *p* < 0.01, *** *p* < 0.001, compared with control cultures or control mice infected with *T. gondii* (two-tailed Student’s *t*-test). U, untreated.

Based on previous studies showing that ω3-PUFAs have immunomodulatory properties in various infectious diseases [9], we examined whether endogenous ω3-PUFAs exhibit host-protective effects against *T. gondii* infection using Fat-1 transgenic mice and Fat-1-derived primary macrophages. To investigate the effect of endogenous ω3-PUFA enrichment, BMDMs were isolated from wild-type (WT) and Fat-1 transgenic mice, infected with a GFP-expressing RH strain of *T. gondii* (GFP-RH) for the indicated periods (Figure 1A–C), and then evaluated for intracellular growth of *T. gondii*. As shown in Figure 1A,B, intracellular proliferation of the parasite was significantly decreased in Fat-1-derived BMDMs when compared to WT BMDMs. Moreover, enrichment of endogenous ω3-PUFAs caused a significant decrease in the number of cells infected with *T. gondii* (Figure 1A,C). We next checked the antiparasitic effects of ω3-PUFAs by analyzing the expression levels of *Toxoplasma*-specific antigens, including p30 membrane protein (TP3) and surface antigen 1 (SAG1) in Fat-1-derived BMDMs. As shown in Figure 1D,E, TP3 protein expression (for D) and *SAG1* mRNA expression (for E) were markedly decreased in Fat-1-derived BMDMs.

To elucidate the functions of endogenous ω3-PUFAs during *T. gondii* infection in vivo, WT or Fat-1 transgenic mice were challenged with the *T. gondii* type II strain ME49 (Figure 1F; 40 cysts/mouse). The cyst burden in brain tissues at 3 weeks after *T. gondii* challenge was significantly lower in Fat-1 transgenic mice than in WT mice (Figure 1F lower panels). Consistent herewith, the *B1* gene copy number were lower in infected brains of Fat-1 transgenic mice than in those of WT mice (Figure 1F, upper panels). Taken together, these results suggested that endogenous ω3-PUFAs play an essential role in host protection against *Toxoplasma* infection.

### 3.2. Autophagy is Required for Docosahexaenoic Acid (DHA)-Mediated Activation of Antiparasitic Responses in Primary Murine Macrophages

**Figure 2:**
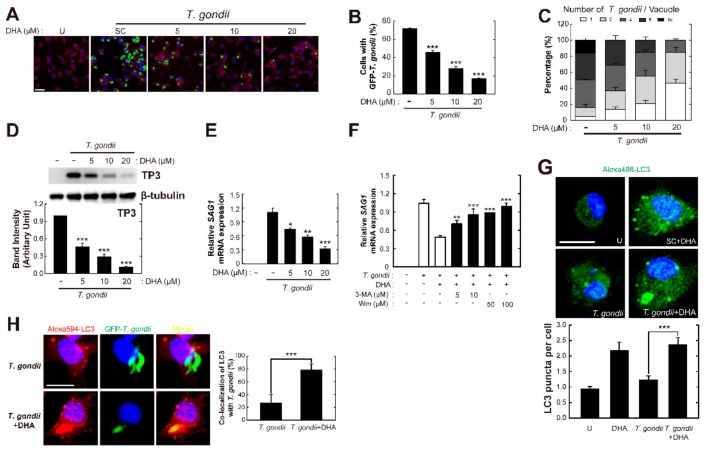
Docosahexaenoic acid (DHA)-induced autophagy activation results in the elimination of intracellular *T. gondii* in primary macrophages. (**A**–**C**) BMDMs were infected with *T. gondii* GFP-RH strain (MOI = 1) then incubated with DHA at indicated concentrations for 18 h. (**A**) The number of GFP-RH strain were analyzed using confocal microscopy. (**B** and **C**) GFP-RH-infected cells (**B**) and GFP-RH strain per vacuole (**C**) were counted. Scale bar = 25 μm. (**D** and **E**) BMDMs were infected with *T. gondii* RH strain (MOI = 1) for 2 h, and then incubated with increasing concentrations of DHA (5, 10, or 20 μM) for 48 h. (**D**) Immunoblot analysis was performed to determine protein expression of TP3 and β-tubulin. A representative gel from three independent experiments with similar results is shown. (top), Densitometry analysis (bottom) (**E**) *SAG1* mRNA expression was evaluated by qPCR analysis (bottom). (**F**) BMDMs were infected with *T. gondii* RH strain (MOI = 1), followed by treatment with 3-methyladenine (3-MA) (5 or 10 μM; 2 h) or wortmannin (WM) (50 or 100 nM; 2 h), and then incubated with DHA (10 μM) for 18 h. *SAG1* mRNA expression was evaluated by qPCR analysis. (**G**) BMDMs were infected with *T. gondii* RH strain (MOI = 1) for 2 h, and then incubated with DHA for 18 h. Immunofluorescence microscopic analysis of LC3 puncta formation (top). Quantitative analysis of LC3 punctate foci per cell (bottom). Each experiment included at least 100 cells scored in 5 random fields. Scale bar: 10 μm. (**H**) BMDMs were infected with *T. gondii* GFP-RH strain (MOI = 3) for 2 h, and then incubated with DHA for 18 h. Cells were observed by confocal microscopy (left); *T. gondii* GFP-RH (green), Alexa 594-conjugated LC3 (red), and DAPI (blue). Quantification of *T. gondii* and LC3 colocalization per cell with 100 internalized *T. gondii* (right). Scale bar: 10 μm. Data in B, C, D, E, and G are means ± SD. Data in G are means ± SEM. Data are representative of three independent experiments. * *p* < 0.05, ** *p* < 0.01, *** *p* < 0.001, compared with solvent control (two-tailed Student’s *t*-test). U, untreated; SC, solvent control.

Previous studies demonstrated that ω3-PUFAs promote autophagy induction in macrophages, thus modulating inflammatory tolerance and inflammasome activation [27,28]. Moreover, exposure of U937 cells to DHA resulted in an enhancement of M2 macrophage polarization through autophagy and p38 MAPK signaling [29]. However, it has not been clarified whether DHA-induced autophagy is essential for host immune defense against *Toxoplasma* infection. To determine optimal concentration of DHA without cytotoxic effects, BMDMs were treated with increasing concentration of DHA and then cell viability were measured with CCK8 assay (Figure S1). As we found that endogenous ω3-PUFAs are essential for host protection against *T. gondii* in vivo and in vitro (Figure 1), we next attempted to investigate the anti-Toxoplasma activity of DHA in primary macrophages (Figure 2A–D). Similar to endogenous ω3-PUFAs, the number of *T. gondii*-infected cells (Figure 2A,B) and the intracellular proliferation rate of *T. gondii* (Figure 2A,C) were significantly reduced upon DHA treatment, in a concentration-dependent manner. Consistent herewith, TP3 protein levels (Figure 2D) and *SAG1* mRNA (Figure 2E) levels were lower in DHA-treated than in non-treated *T. gondii*-infected BMDMs.

Next, we sought to examine the role of autophagy in the effect of DHA in restricting intracellular survival of *T. gondii*. As shown in Figure 2F, pretreatment with an autophagy inhibitor (3-MA or wortmannin (WM)) resulted in significant inhibition of the anti-*Toxoplasma* activity of DHA in *T. gondii*-infected BMDMs. Furthermore, DHA stimulation of *T. gondii*-infected BMDMs induced LC3 aggregate formation (Figure 2G) and DHA treatment significantly increased intracellular colocalization of LC3-positive autophagic vacuoles with *T. gondii*-containing PVs in BMDMs as compared to non-treated infected cells (Figure 2H). These data suggested that autophagy activation is required for DHA-mediated antiparasitic responses in primary macrophages.

### 3.3. DHA Induces Autophagosome and Autolysosome Formation in Primary Murine Macrophages

**Figure 3:**
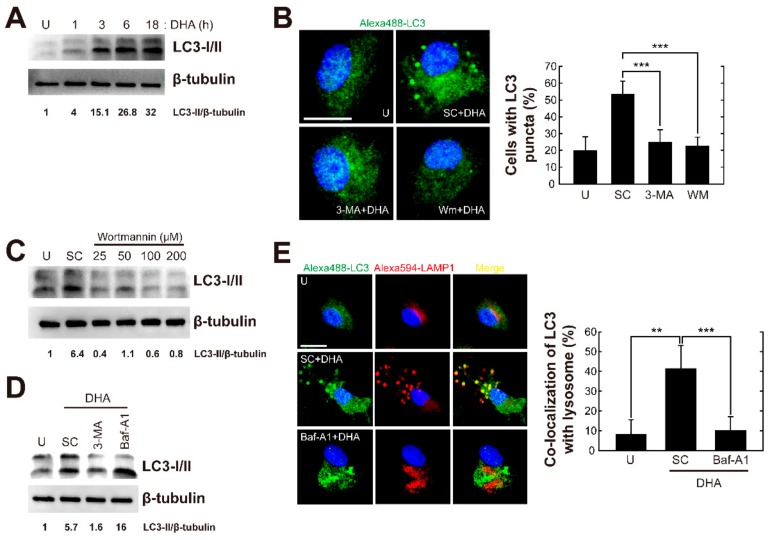
DHA induces autophagy activation in primary macrophages. (**A**) BMDMs were treated with DHA (10 μM) for the indicated time periods and subjected to immunoblot analysis of LC3 and β-tubulin. (**B**–**E**) BMDMs were treated with 3-MA (10 μM), Baf-A1 (100 nM), or increasing concentrations of WM (25, 50, 100, or 200 nM), and then incubated with DHA for 18 h. (**B**) Alexa 488-conjugated LC3 (green) and DAPI (blue) were detected by confocal microscopy. Merged signals of LC3 puncta formation and DAPI (left). Quantitative analysis of the percentage of cells with LC3 puncta (right). Scale bar: 10 μm. (**C** and **D**) Cell lysates were subjected to immunoblot analysis of LC3 and β-tubulin. (**E**) Alexa 488-conjugated LC3 (green) and Alexa 594-conjugated lysosome-associated membrane glycoprotein 1 (LAMP1, red) and DAPI were detected by confocal microscopy. Merged signals of LC3, LAMP1, and DAPI (left). Quantitative analysis of cells showing colocalization of LC3 with LAMP1 (right). Scale bar: 10 μm. Each immunofluorescence microscopic experiment included at least 100 cells scored in 5 random fields. Data are representative of three independent experiments and are presented as means ± SD. ** *p* < 0.01, *** *p* < 0.001 (two-tailed Student’s t-test). U, untreated; SC, solvent control.

Autophagy reportedly contributes to DHA-mediated cytotoxicity in diverse cancer cells [30,31]. To determine whether DHA treatment activates autophagic responses in macrophages, we evaluated the number of cells with microtubule-associated protein 1 light chain 3 (LC3), an essential autophagy effector. Protein levels of LC3-phosphatidylethanolamine conjugate (LC3-II) were gradually upregulated as of 3 h after DHA treatment and peaked 6–18 h in primary murine macrophages. The ratio of LC3-II to LC3-I was also increased in BMDMs treated with DHA (Figure 3A). As shown in Figure 3B, DHA treatment resulted in an increase in LC3 aggregates in BMDMs, and this increase was significantly attenuated in cells pretreated with autophagy inhibitor 3-MA or WM. Consistent with the immunostaining results, elevated levels of LC3-II protein induced by DHA were significantly attenuated by pretreatment with WM (Figure 3C) or 3-MA (Figure 3D).

During autophagy, cytoplasmic components engulfed in autophagosomes are degraded by autolysosomes formed through fusion with lysosomes [18]. Therefore, we examined whether DHA stimulation leads to autophagosome-lysosome fusion, using confocal microscopy. As shown in Figure 3E, stimulation of BMDMs with DHA resulted in the colocalization of LC3 (an autophagosomal marker) and LAMP-1, a lysosomal marker, and this fusion was attenuated after pretreatment with the vascular H+-ATPase inhibitor bafilomycin A1 (Baf-A1). In addition, DHA-induced LC3-II protein expression was enhanced in BMDMs pretreated with Baf-A1 (Figure 3D). Collectively, these data indicated that DHA triggers autophagy activation in primary murine macrophages.

### 3.4. Macrophages Enriched in ω3-PUFAs Exhibit Increased Autophagy Activation in Response to T. gondii Infection

**Figure 4:**
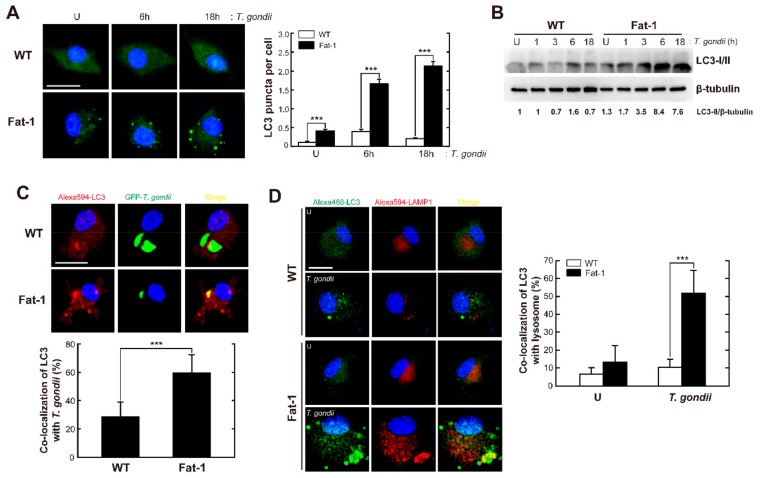
Endogenous ω-3 PUFA-induced autophagy activation leads to phagosomal maturation in macrophages. (**A** and **B**) BMDMs from WT and Fat-1 transgenic mice were infected with *T. gondii* RH strain (MOI = 1) for the indicated time periods. (**A**) Immunofluorescence microscopic analysis of LC3 puncta formation (left). Quantitative analysis of LC3 punctate foci per cell (right). Scale bar: 10 μm. (**B**) Cell lysates were subjected to immunoblot analysis of LC3 and β-tubulin. (**C**) BMDMs from WT and Fat-1 transgenic mice were infected with *T. gondii* GFP-RH strain (MOI = 3) for 18 h. Immunofluorescence microscopic analysis of colocalization of LC3-positive autophagic vacuoles with GFP-RH strains (left). Quantification of *T. gondii* and LC3 colocalization per cell with 100 internalized *T. gondii* (right). Scale bar: 10 μm. (**D**) BMDMs from WT and Fat-1 transgenic mice were infected with *T. gondii* RH strain (MOI = 1) for 18 h. Immunofluorescence microscopic analysis of colocalization of LC3 and LAMP1 (left)**.** Quantitative analysis of cells showing colocalization of LC3 and LAMP1 (right). Scale bar: 10 μm. Each immunofluorescence microscopic experiment included at least 100 cells scored in 5 random fields. Data in A are means ± SEM. Data in C and D are means ± SD. Data are representative of three independent experiments. *** *p* < 0.001 (two-tailed Student’s *t*-test). U, untreated.

It was recently reported that endogenous ω3-PUFA accumulation in Fat-1 transgenic mice prevented streptozotocin-induced pancreatic β-cell death through the activation of autophagy [32]. To evaluate the effect of endogenous ω3-PUFAs on autophagic responses during *T. gondii* infection, BMDMs isolated from WT or Fat-1 transgenic mice were infected with *T. gondii* RH strain for the indicated time periods. Similar to our observations in DHA-treated primary macrophages, LC3 aggregate formation (Figure 4A) and LC3-II protein expression (Figure 4B) were higher in Fat-1-expressing than in WT BMDMs, not only at the basal level, but also after *T. gondii* infection. Furthermore, colocalization of LC3-positive autophagic vacuoles with GFP-RH (Figure 4C) or lysosomes (Figure 4D) was stronger in Fat-1-expressing than in WT BMDMs, suggesting that the enrichment of endogenous ω3-PUFAs led to the induction of autophagosome formation and, consequently, fusion with lysosomes.

### 3.5. CaMKKβ-dependent, Not LKB1-Dependent AMP-Activated Protein Kinase (AMPK) Signaling Is Responsible for Endogenous ω3-PUFA-Mediated Intracellular Growth Inhibition of T. gondii in Primary Macrophages

**Figure 5:**
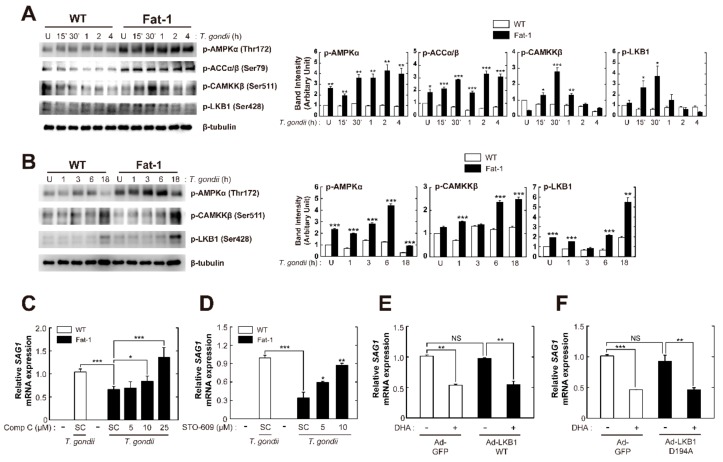
CaMKKβ/AMPK activation plays an essential role in endogenous ω-3 PUFA-induced antiparasitic responses against *Toxoplasma* infection in BMDMs. (**A**,**B**) BMDMs from WT and Fat-1 transgenic mice were infected with *T. gondii* RH strain (MOI = 1) for the indicated time periods. (A) Immunoblot analysis of phosphorylated forms of AMPKα, ACCα/β, CAMKKβ, LKB1, and β-tubulin. A representative gel from three independent experiments with similar results is shown. (left), Densitometry analysis (right) (**B**) Immunoblot analysis of phosphorylated forms of AMPKα, CAMKKβ, LKB1, and β-tubulin. A representative gel from three independent experiments with similar results is shown. (top), Densitometry analysis (bottom) (**C**,**D**) BMDMs from Fat-1 transgenic mice were incubated with increasing concentrations of the AMPK inhibitor Compound C (5, 10, or 25 μM) or the CaMKK2 inhibitor STO-609 (5 or 10 μM), and then infected with *T. gondii* RH strain (MOI = 1) for 18 h. *SAG1* mRNA expression was evaluated by Real-Time Quantitative PCR analysis. (**E**,**F**) BMDMs transduced for 36 h with adenovirus expressing GFP (Ad-GFP), WT LKB1 (Ad-LKB1 WT), or LKB1 D194A mutant (Ad-LKB1 D194A), at a MOI of 10, were infected with *T. gondii* RH strain (MOI = 1) for 2 h, and then incubated with DHA (10 μM) for 18 h. *SAG1* mRNA expression was evaluated by RT-qPCR analysis. Data are representative of three independent experiments and are presented as means ± SD. * *p* < 0.05, ** *p* < 0.01, *** *p* < 0.001 (two-tailed Student’s *t*-test). NS, not significant; U, untreated; SC, solvent control.

Growing evidence shows that AMPK is a key modulator of ω3-PUFA functions in diverse physiological conditions, such as autophagy, cell death, and stress responses [33,34,35,36]. Fat-1-derived macrophages exhibited increased phosphorylation of AMPKα and Acetyl-CoA carboxylase (ACC)α/β in normal condition and in the early phase (15 min to 4 h) of *T. gondii* infection when compared with WT macrophages. Moreover, phosphorylation of CaMKKβ and liver kinase B1 (LKB1), protein kinases upstream of AMPK, was enhanced in Fat-1-derived macrophages with infected *T. gondii* (Figure 5A). We then evaluated phosphorylation of AMPK, CaMKKβ, and LKB1 in the late phase of *T. gondii* infection. Notably, *T. gondii*-induced phosphorylation of AMPK, CaMKKβ, and LKB1 was obviously increased at 18 h after infection in Fat-1-derived macrophages compared to WT macrophages (Figure 5B). We next investigated whether AMPK signaling is required for endogenous ω3-PUFA-induced antiparasitic responses in primary macrophages. Pretreatment of *T. gondii*-infected Fat-1-derived macrophages with compound C (Comp C), a selective AMPK inhibitor, significantly increased *SAG1* mRNA expression in a concentration-dependent manner (Figure 5C). Upon pretreatment with STO-609, a selective inhibitor of CaMKK, the survival rate of intracellular *T. gondii* in Fat-1-derived macrophages increased to the level observed in WT cells (Figure 5D).

As LKB1 is an essential kinase upstream of AMPK [37], we examined the role of LKB1 in the enhanced antiparasitic responses in Fat-1-derived macrophages. As shown in Figure 5E, overexpression of WT LKB1 (Ad-LKB1 WT) did not affect *SAG1* mRNA expression in both basal and DHA-stimulated conditions in BMDMs. Moreover, adenoviral transduction with a catalytically inactive form of LKB1 (Ad-LKB1 D194A; a mutation of aspartic acid residue 194 to alanine) did not alter DHA-induced antiparasitic responses in BMDMs (Figure 5F). These results suggested that the ω3-PUFA-mediated antiparasitic response is activated via CaMKKβ-dependent AMPK signaling.

### 3.6. AMPK Signaling is Required for DHA-Mediated Activation of Autophagy and Antiparasitic Responses in Bone Marrow-Derived Macrophages (BMDMs)

**Figure 6:**
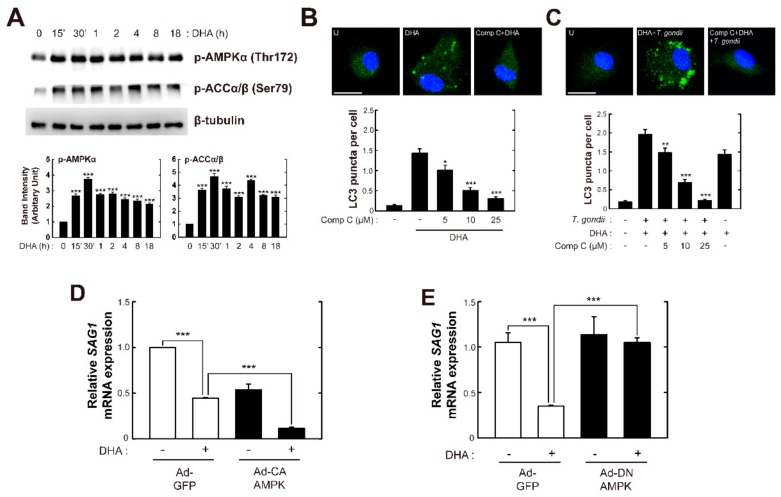
DHA treatment increases host defenses against *T. gondii* infection via AMPK signaling in primary macrophages. (**A**) BMDMs were treated with DHA (10 μM) for the indicated periods. Immunoblot analysis of phosphorylated forms of AMPKα, p-ACCα/β, and β-tubulin. A representative gel from three independent experiments with similar results is shown. (top), Densitometry analysis (bottom) (**B**,**C**) Immunofluorescence microscopic analysis of LC3 puncta formation (top). Quantitative analysis of LC3 punctate foci per cell (bottom). (**B**) BMDMs were treated with Comp C (5, 10, or 25 μM), and then incubated with DHA for 18 h. (**C**) BMDMs were infected with *T. gondii* RH strain (MOI = 1), treated with Comp C (5, 10, or 25 μM), and then incubated with DHA for 18 h. For each experiment, at least 100 cells were scored in 5 random fields. Scale bar: 10 μm. (**D**,**E**) BMDMs transduced for 36 h with adenovirus expressing GFP (Ad-GFP), constitutively active AMPK (Ad-CA), or dominant negative AMPK (Ad-DN), at a MOI of 10, were infected with *T. gondii* RH strain (MOI = 1) for 2 h, and then incubated with DHA for 18 h. *SAG1* mRNA expression was evaluated by RT-qPCR analysis. Data in B and C are means ± SEM. Data in D and E are means ± SD. Data are representative of three independent experiments. * *p* < 0.05, ** *p* < 0.01, *** *p* < 0.001, compared with solvent control (two-tailed Student’s *t*-test). U, untreated; SC, solvent control.

Numerous studies have demonstrated that AMPK plays an essential regulatory role in the autophagy pathway via modulating ULK1 complex and mTORC1 complex activities [19,32]. Indeed, Liu et al. reported that CD40 stimulates autophagy-mediated elimination of *T. gondii* in macrophages via CaMKKβ/AMPK-mediated ULK1 phosphorylation [38]. We previously reported that DHA-mediated autophagy promotes apoptosis through AMPK singling in cancer cells harboring WT p53 [30]. Because AMPK signaling is upregulated in Fat-1-derived macrophages infected with *T. gondii* (Figure 5A,B), we next examined whether AMPK is involved in autophagy induction in DHA-treated primary macrophages. DHA stimulation rapidly (within 15 min) induced activation of AMPKα and ACCα/β, which was sustained up to 18 h (Figure 6A). Importantly, the pretreatment of BMDMs with Comp C significantly attenuated DHA-mediated autophagosome formation in a dose-dependent manner (Figure 6B). Similarly, the DHA-mediated increase in LC3 aggregates in BMDMs infected with *T. gondii* was attenuated in the presence of Comp C (Figure 6C).

We next investigated whether AMPK signaling is involved in the elimination of *T. gondii* in macrophages. Overexpression of a constitutively active AMPK using an adenoviral transduction system reduced *SAG1* mRNA expression in both basal and DHA-stimulated conditions in BMDMs (Figure 6D). However, adenoviral transduction of dominant-negative AMPK attenuated the *T. gondii*-killing effect of DHA, although there was no specific change in the basal condition (Figure 6E). Collectively, these results suggested that AMPK signaling is required for DHA-induced autophagosome formation and the activation of antiparasitic responses.

## 4. Discussion

*T. gondii* has a complicated life cycle with sexual reproduction in definitive host and asexual replication in any warm-blooded animal. In the human and mouse, two different forms including tachyzoites and bradyzoites can be distributed into diverse organs and tissues [11]. In this study, we found that the accumulation of endogenous ω3-PUFAs in Fat-1-derived primary macrophages significantly attenuated intracellular parasitic growth (RH strain of tachyzoites). Consistent herewith, Fat-1 transgenic mice infected with an avirulent strain of *T. gondii* (ME49 strain of bradyzoites) showed the reduced protozoan load in the brain when compared with infected WT littermates. This study is the first to report that ω3-PUFAs play an important role in controlling *Toxoplasma* infection in vivo and in vitro, and that they are not essential nutrients for parasite growth. Nevertheless, it is still investigated whether ω3-PUFAs is also beneficial in human innate cells including monocytes and macrophages or for protecting different strains of *T. gondii*. Thus, further experiments are required to solve these questions.

Protozoan parasites belonging to the phylum Apicomplexa are known to be critical causal agents of various human diseases, including toxoplasmosis, malaria, and cryptosporidiosis. These parasites are capable of surviving and proliferating within diverse cells by depleting host nutrients; therefore, previous studies have focused on identifying the strategies these parasites employ to utilize host nutrients. It is also well known that nutrient status is closely associated with immune homeostasis and activation. Moreover, diverse nutrient- and bacteria-derived factors, especially fatty acids, mediate the innate and acquired immune systems through Toll-like receptor pathways [39]. As such, various lipid components act as either bacterial energy sources or immune-activating factors, depending on host conditions, infectious agents, and nutrient sources, which has led to controversial results in this respect. In *Toxoplasma* infection, lipid biosynthesis via both salvage and de novo pathways plays an important role in intracellular growth and pathogenesis, indicating the administration of ω3-PUFAs may be detrimental [40,41,42]. On the other hand, increasing evidence suggested that ω3-PUFAs may be attractive therapeutic candidates to improve inflammatory bowel diseases via regulating excessive inflammation and immune responses [43]. It is interesting issue whether ω3-PUFAs is essential for the maintain the gut microbiota and mucosal homeostasis regarding *T. gondii* infects individual humans through the ingestion of contaminated feline fecal material, food, and water. Although studies suggested that immunomodulatory functions of ω3-PUFAs may be used as a therapeutic strategy in infectious and inflammatory diseases [9], the roles of ω3-PUFAs in response to *T. gondii* remained unclear.

Regarding the function of ω3-PUFAs in response to *Mycobacterium tuberculosis* (Mtb), a facultative intracellular pathogen that causes tuberculosis, controversial results have been reported. An early study demonstrated that treatment of J774A.1 macrophage with EPA resulted in enhanced intracellular growth of Mtb, whereas a mice fed a diet enriched in ω3-PUFAs exhibited significantly reduced bacterial burden in the lungs and spleens at 21 and 63 days after intranasal inoculation [44]. Only a month later, McFarland et al. reported that consumption of dietary ω3-PUFAs resulted in increased bacterial burden in the lungs of guinea pigs infected with Mtb when compared with dietary ω6-PUFA-fed guinea pigs [45]. In a subsequent study, Fat-1-derived primary macrophages and mice exhibited impaired resistance to Mtb infection [8]. Although the experimental conditions used in these studies are different, these results highlight the need for a cautious approach when investigating the role of ω3-PUFAs in infectious diseases. Inspired by these conflicting results, we examined whether DHA has the ability to control *T. gondii* infection, and we found that, similar to our observations in Fat-1-derived BMDMs, DHA stimulation effectively attenuated intracellular growth of *T. gondii* in BMDMs.

Increasing evidence indicates that autophagy, a self-digestive lysosomal degradation pathway, is essential for the elimination of intracellular pathogens via a specialized selective process called xenophagy [19,46]. In *Toxoplasma* infection, canonical [47] or non-canonical autophagy [48,49] is involved in the restriction of intracellular *T. gondii*. Previous studies have focused on the roles of autophagy in ω3-PUFA-mediated attenuation of tumor cell growth [31,47] or inflammasome activation in macrophages [27,50]. However, it was not revealed whether ω3-PUFAs activate autophagy and, consequently, anti-parasitic activity in primary murine macrophages. In this study, we found that DHA induced autophagy in primary macrophages. Moreover, DHA-mediated autophagy was required for controlling intracellular *T. gondii* growth. Consistent with these findings, autophagy induction and autophagolysosome formation in response to *T. gondii* infection were enhanced through endogenous ω3-PUFA accumulation in Fat-1-derived macrophages as compared with WT cells. Our results strongly suggested that autophagy activation by DHA and/or endogenous ω3-PUFAs plays an important role in controlling *T. gondii* growth.

AMPK is tightly regulated by upstream kinases, such as LKB1 and CaMKKβ, and plays a pivotal role in regulating cellular energy homeostasis, thereby providing an attractive therapeutic target for metabolic diseases and cancer [51]. In addition, AMPK is involved in the activation of host defense against diverse infectious agents, such as viruses, bacteria, and parasites, via regulating innate immune and inflammatory responses [52]. A recent study demonstrated that CaMKKβ/AMPK signaling is required for CD40-induced autophagic killing of *T. gondii* [38]. Thus, we examined the role of AMPK signaling in ω3-PUFA-mediated autophagy and antiparasitic activity. The results showed that DHA-mediated autophagy was effectively attenuated by the AMPK-specific inhibitor Comp C in naïve as well as *T. gondii*-infected BMDMs. DHA-mediated killing of *T. gondii* was enhanced in cells overexpressing constitutively active AMPK, whereas it disappeared upon overexpression of dominant-negative AMPK. Moreover, the parasitic killing observed in Fat-1-expressing macrophages was suppressed in the presence of Comp C or STO609, indicating that CaMKKβ/AMPK-mediated autophagy is crucial for the host-protective effect of ω3-PUFAs against *T. gondii* infection.

## 5. Conclusions

**Figure 7:**
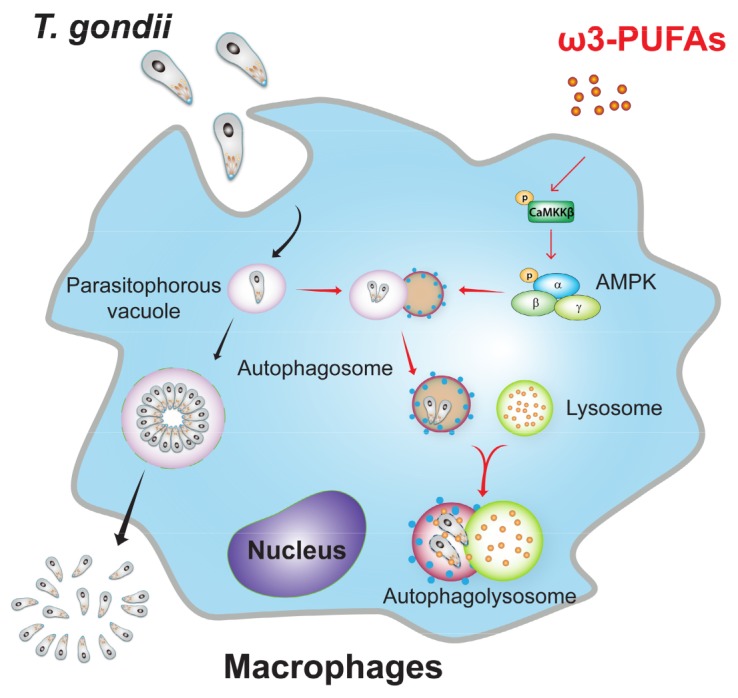
Schematic model representing the novel activities of ω3-PUFAs against *T. gondii* infection. Endogenous enriched ω3-PUFAs or the treatment of DHA induced CaMKKβ/AMPK-dependent autophagy activation in primary murine macrophages, leading to activation of host protective responses against *T. gondii* infection.

Collectively, our findings suggest that ω3-PUFAs restrict the survival and proliferation of the intracellular protozoon *T. gondii* via AMPK-dependent autophagy activation (Figure 7). ω3-PUFAs, which are often consumed as a nutritional supplement, are considered to be an optimal therapeutic candidate to prevent toxoplasmosis. Additional studies are necessary to investigate an optimal treatment regimen for toxoplasmosis via the most effective combination with other candidate drugs and to evaluate the clinical significance of ω3-PUFAs in *Toxoplasma* infection.

## Supplementary Materials

The following are available online at https://www.mdpi.com/2072-6643/11/9/2137/s1, Figure S1: Cytotoxic effects of DHA in BMDMs. BMDMs were treated with different concentrations of DHA (5–500 μM) for 18 h, and cell viability was evaluated using CCK-8 assay. Quantitative data for cell viability are representative of three independent experiments and are presented as means ± SD. ** *p* < 0.01, *** *p* < 0.001 (two-tailed Student’s *t*-test), compared with SC-treated cells. SC, vehicle control (0.01% DMSO).
