# Supplementary material for: Omega-3 Polyunsaturated Fatty Acids Prevent Toxoplasma gondii Infection by Inducing Autophagy via AMPK Activation
Source: Nutrients. 2019 Sep 6;11(9):2137. doi: 10.3390/nu11092137 (PMC6771136; doi:10.3390/nu11092137)

Omega-3 Polyunsaturated Fatty Acids Prevent *Toxoplasma gondii* Infection by Inducing Autophagy via the AMPK activation.

Running title: ω3-PUFAs restrict *T. gondii* growth via autophagy

Jae-Won Choi[1,2,3¶], Jina Lee[1,2,3¶], Jae-Hyung Lee[1,2,3], Byung-Joon Park[1,2,3], Eun Jin Lee[1,2,3], Soyeon Shin[4], Guang-Ho Cha[1,2], Young-Ha Lee[1,2], Kyu Lim[4*], Jae-Min Yuk[1,2,3*]

[1]Department of Infection Biology, College of Medicine, Chungnam National University, Daejeon, Korea; [2]Department of Medical Science, College of Medicine, Chungnam National University, Daejeon, Korea; [3]Infection Control Convergence Research Center, College of Medicine, Chungnam National University, Daejeon, Korea; [4]Department of Biochemistry, College of Medicine, Chungnam National University, Daejeon, Korea.

Supplemental Materials and methods

Cell count kit (CCK) 8 assay

The cytotoxicity effects of herbal extracts on BMDMs were determined using CCK8 assay (Dojindo Molecular Technologies, CK04-11) according to manufacturer’s protocol. Briefly, BMDMs were seeded in 96well plates and differentiated with M-CSF for 5 days as described in cell preparation. Cells were replaced with serum-free media and then incubated with various DHA for 18 hours. Then, 10 μl of the CCK-8 solution was added and incubated for 1 hours at 37°C. Absorbance was measured at 450 nm using a microplate reader (SPECTRO star Nano, BMG Labtech, Ortenberg, Germany). The Optical density (OD) of supernatants for each treated well was calculated by dividing the average OD from control wells and quantified values of cell viability were obtained.

Supplemental Figures

Fig S1. Cytotoxic effects of DHA in BMDMs. BMDMs were treated with different concentrations of DHA (5 - 500 μM) for 18 hours, and cell viability was evaluated using CCK-8 assay. Quantitative data for cell viability are representative of three independent experiments and are presented as means ± SD. $^{**}P < 0.01$, $^{***}P < 0.001$ (two-tailed Student's t-test), compared with SC-treated cells. SC, vehicle control (0.01% DMSO).

Figure S1. Choi *et al*.

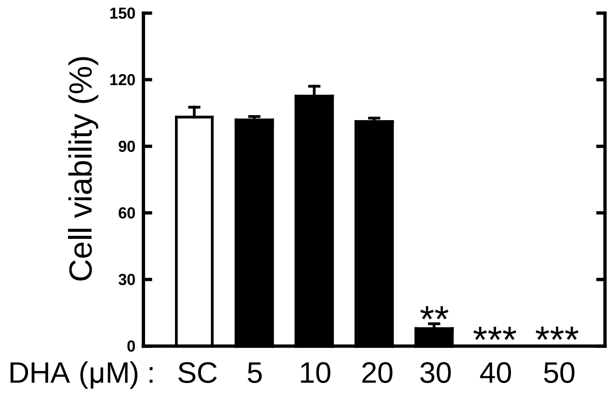

Supplement: Supplementary file 1 [file nutrients-11-02137-s001.pdf]
